# Liquid biopsy: Exosomal microRNAs as novel diagnostic and prognostic biomarkers in cancer

**DOI:** 10.1186/s12943-022-01525-9

**Published:** 2022-02-16

**Authors:** K. Auxzilia Preethi, Sushmaa Chandralekha Selvakumar, Kehinde Ross, Selvaraj Jayaraman, Deusdedit Tusubira, Durairaj Sekar

**Affiliations:** 1 Centre for Cellular and Molecular Research, Saveetha Dental College and Hospital, Saveetha Institute of Medical and Technical Sciences, Saveetha University, Chennai, Tamil Nadu 600077 India; 2 School of Pharmacy and Biomolecular Sciences, Liverpool John Moores University, Liverpool, UK; 3 Department of Biochemistry, Saveetha Dental College and Hospital, Saveetha Institute of Medical and Technical Sciences (SIMATS), Saveetha University, Chennai, 600077 India; 4 Biochemistry Department, Mbarara University of Science and Technology, Mbarara, Uganda

**Keywords:** Liquid biopsy, Cancer, Exosomal miRNAs, Biomarkers, Non-invasive diagnosis

## Abstract

**Background:**

Detecting cancer at an early stage before clinical manifestation could be an effective strategy to decrease cancer mortality. Thus, identifying liquid biopsy biomarkers with high efficacy could be a promising approach for non-invasive diagnosis of cancer.

**Main text:**

Liquid biopsies are increasingly used as a supplement to biopsy, as it enables disease progression to be detected months before clinical and radiographic confirmation. Many bodily fluids contain exosomal microRNAs (miRNAs) which could provide a new class of biomarkers for early and minimally invasive cancer diagnosis due to the stability of miRNAs in exosomes. In this review, we mainly focused on the exosomal miRNAs (liquid biopsy) as biomarkers in the diagnosis and prognosis of various cancers.

**Conclusion:**

Exosomal miRNAs can be used as diagnostic and prognosis biomarkers that provide unique insights and a more dynamic perspective of the progression and therapeutic responses in various malignancies. Therefore, the development of novel and more sensitive technologies that exploit exosomal miRNAs should be a priority for cancer management.

## Introduction

Cancer is the major cause of the death globally, and the number of cancer diagnoses and deaths are predicted to rise significantly as populations grow, age, and adopt cancer-risking lifestyle choices [1]. According to World Health Organization, cancer is the second major cause of mortality worldwide, accounting for approximately 10 million deaths and 19.3 million new cancer cases in 2020 [2]. Cancer treatment currently includes surgery, radiation, and the use of chemotherapeutic drugs, which commonly destroy healthy cells and cause toxicity in patients [3].

**Fig. 1:**
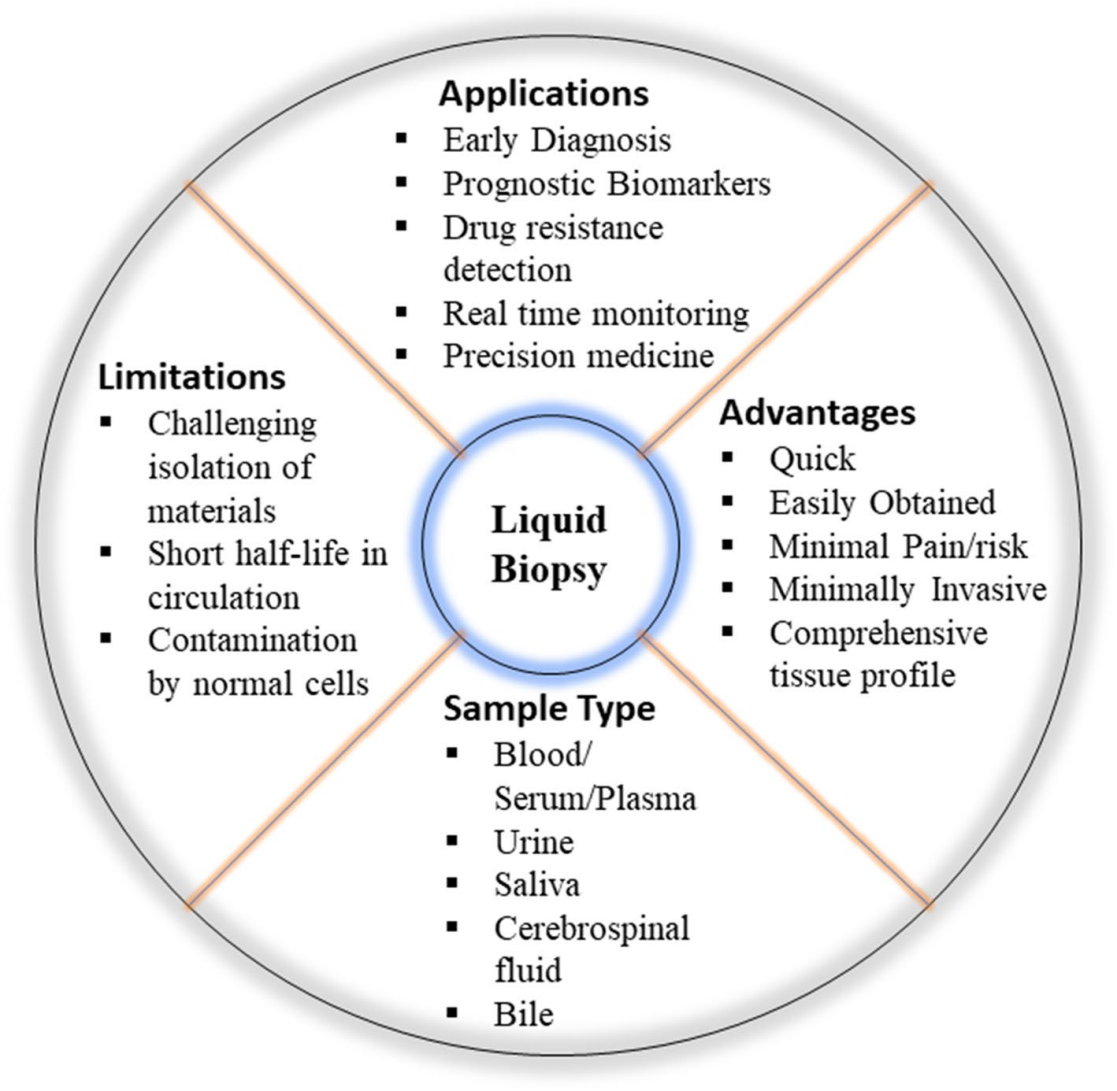
Representing the overview of liquid biopsy

Tumor biopsy is an invasive procedure widely used for cancer diagnosis. This procedure poses significant risks to patients and may put their lives in danger. Furthermore, biopsies have the capability to destroy the surrounding tissue, promoting cancer metastasis and progression [4]. Liquid biopsy is a multimodal diagnostic approach in clinical oncology that is becoming more popular as it enables the identification, investigation, and monitoring of cancer in body fluids such as blood, serum, plasma or urine [5]. Some of the biological components found in the blood are platelets, circulating cells, miRNA, extracellular vesicles, mRNA, cell-free DNA (cf-DNA) and proteins [6]. For example, a percentage of cf-DNA is released from cancer patients’ blood by tumor cells via apoptosis, necrosis or active release, and this DNA is known as circulating tumor DNA (ctDNA). Tumor-specific mutations in the ctDNA sequence may serve as novel form of cancer biomarker, allowing cancer patients to be distinguished from a group of healthy people [6]. Some of the advantages of liquid biopsy over tumor biopsy are more easily accessible, less painful, and more comprehensive for evaluating tumor heterogeneity because all tumor sites are released into the blood [6]. Thus, identifying liquid biopsy biomarkers with high efficacy could be a promising approach for non-invasive diagnosis of cancer before clinical manifestation of the disease, which is considered as an effective strategy to lower cancer mortality. Figure 1 representing overview of liquid biopsy.

Exosomes increasingly referred to as small extracellular vesicles [7] have captivated the interest of biomedical researchers particularly because they carry microRNAs (miRNAs) associated with cancer processes such as angiogenesis and metastasis [8]. As reported, with a certain number of cancer-specific biomarkers, tumor cells may produce more exosomes than normal ones. Thus, exosomes have been recognised as the most promising and basic indicators for the diagnosis of patients because they are easily accessible and preserve stability in *invitro*. Exosomal miRNAs have been discovered in all human physiological fluids, including plasma, serum, urine, saliva, bile, breast milk, and cerebrospinal fluids, and are being exploited as non-invasive tumour indicators [9]. Because of the prevalence of exosomes in such fluids and the stability of miRNAs, exosomal miRNAs may provide a unique class of biomarkers for early and less invasive cancer diagnosis. This review appraises recent studies on potential of exosomal miRNAs as diagnostic and prognostic biomarkers in cancers, including like lung, breast, prostate, oral squamous cell carcinoma (OSCC) and colorectal cancer (CRC).

### Biogenesis of exosomes

**Fig. 2:**
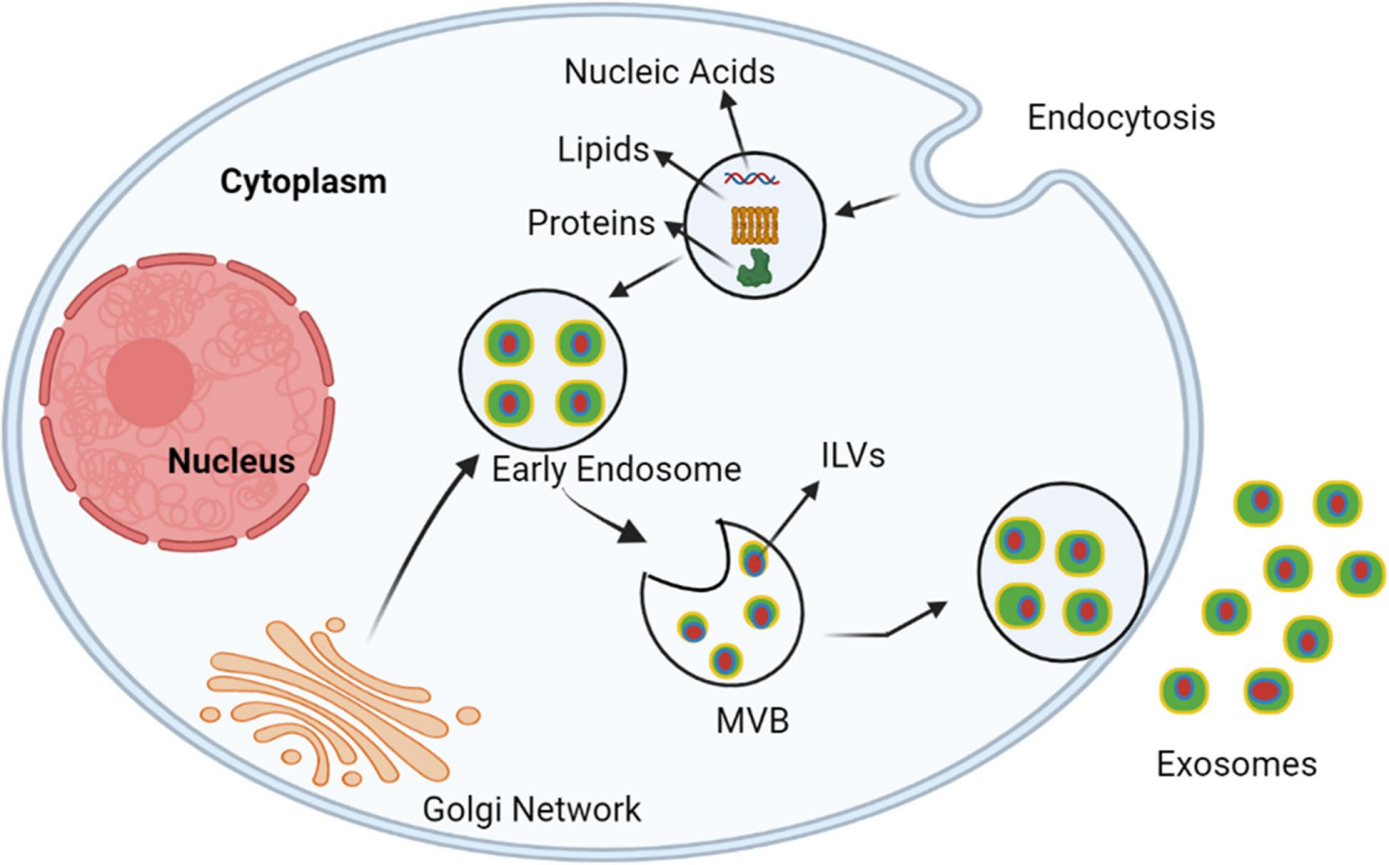
Representing the biogenesis of Exosomes. Exosomes are produced by intraluminal vesicles (ILVs). The cargoes like nucleic acids, lipids and proteins are absorbed by the cells and are carried to early endosomes via the endocytotic pathway. Multivesicular bodies (MVBs) are formed by the maturation of early endosomes. Nucleic acids like miRNAs, DNAs, and RNAs, proteins like cytoplasmic proteins, tetraspanins, and membrane receptors, and lipids like ceramides and cholesterol are all integrated into exosomes during the ILV production process. Finally, exosomes are released into the extracellular space when MVBs fuse with cellular membranes

Exosomes are endosomal-derived lipid bilayers with diameter of 40–100 nm that contain a wide range of bioactive molecules such as nucleic acids, lipids and proteins [10]. Exosomes are secreted from all cell types namely immune cells, tumor cells and epithelial cells [11, 12] and are also found in biological fluids like plasma, serum, urine, saliva, bile, breast milk and cerebrospinal fluids [13]. In relative to healthy proliferating cells, tumour cells generate an excessive number of exosomes [14]. Exosomes carry cargos (proteins, lipids and nucleic acids) from donor to destination cells via target cell membrane fusion, and hence play a role in cell-cell communication and the regulation of numerous physiological functions [15]. Exosome biogenesis is a two-step process. At first, the development of early exosomes begins with the manifestation of the cell membrane. The inward introversion of endosomal membranes results in the formation of multiple intraluminal vesicles (ILVs), which contributes to the creation of multivesicular bodies (MVBs) [16]. Cytosolic constituents like as nucleic acids, proteins, and lipids can be processed into ILVs during this mechanism. MVBs merge with the plasma membrane, delivering ILVs into the extracellular environment [17]. Exosome biogenesis has been linked to a number of causes. The endosomal sorting complex required for transport (ESCRT) machinery is involved in sorting cargo into the ILVs, which are released from the MVBs as exosomes [18]. ESCRT has four main functions namely the assembling and collecting of cargoes (lipids, proteins and nucleic acids) is the responsibility of ESCRT-0. ESCRT-I and -II have the ability to stimulate the creation of a membrane bud, which confines cargoes. The ESCRT-III complex governs membrane scission from the bud’s cytoplasmic side. Cargoes are captured in ILVs after scission, while ESCR-III remains on the outside of the residual membrane until it is regenerated [19]. In addition, supplementary proteins including ALG-2-interacting protein X (ALIX) and tumour susceptibility gene 101 (TSG101) are important in cargo wrapping and exosome formation [20]. Figure 2 representing the biogenesis of exosomes.

### Sorting of miRNAs into exosomes

Exosomal cargo is a new area of research that could help us in understanding the molecular mechanisms behind exosomes’ participation in cancer progression. Lipids and proteins make up the majority of exosome membranes, while nucleic acids such as messenger RNAs (mRNAs), non-coding RNAs (ncRNAs) and miRNAs are found in the exosomal lumen [21, 22]. Because of regulatory involvement in gene expression, miRNAs have gained the most attention among these molecules. miRNAs are the class of small ncRNAs with 19–25 nucleotides in length and play a major role in gene expression studies at post-transcriptional level [23]. Furthermore, miRNAs and their target genes form complex regulatory networks that helps a wide range of biological processes like cell proliferation, differentiation, and death [24]. Exosomes containing miRNAs can circulate with their associated vehicles to reach neighbouring and distant cells. Exosomal miRNAs perform a functional role after being transported into recipient cells [25].

**Fig. 3:**
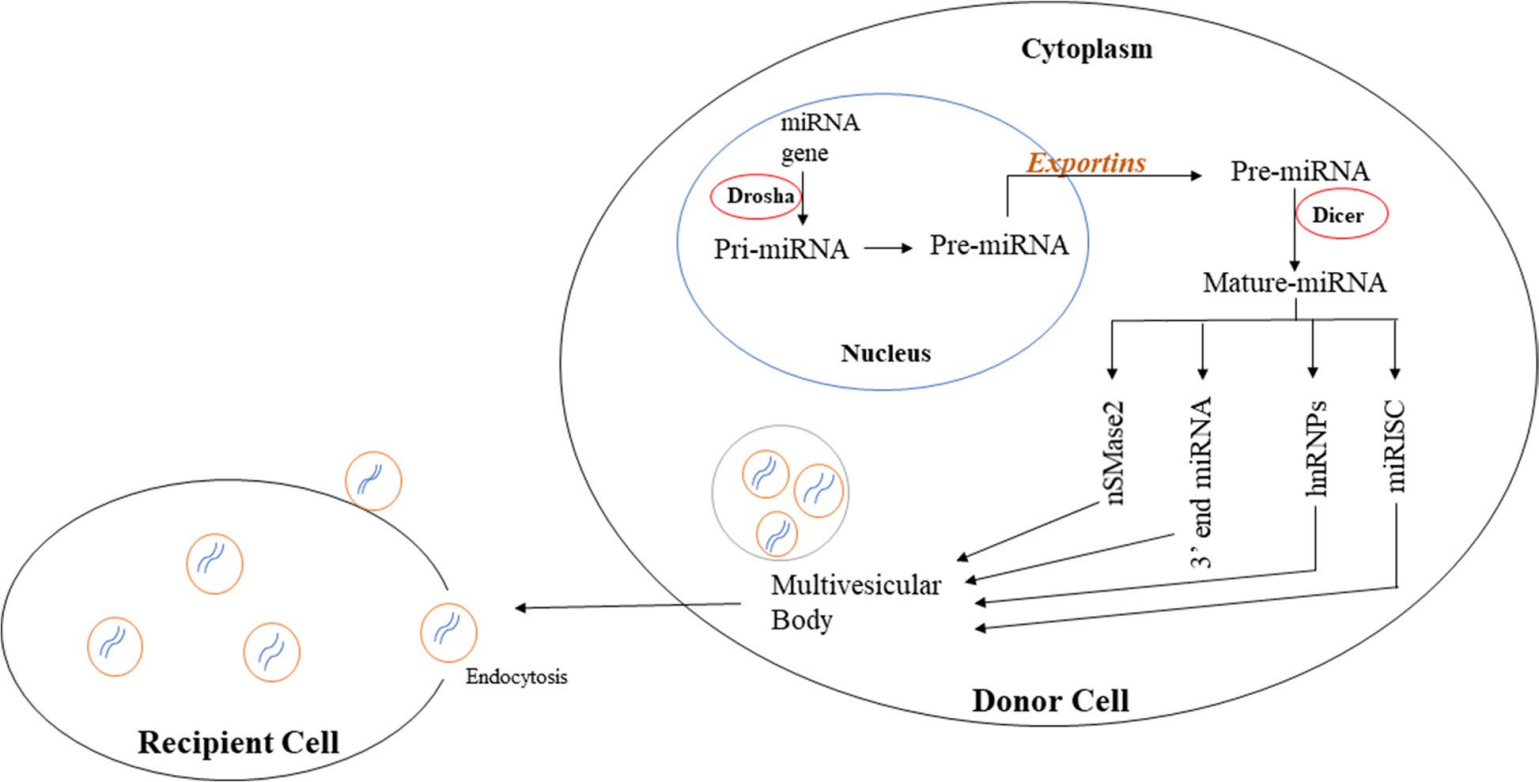
Representing sorting mechanism of miRNAs into exosomes. miRNA genes are transcribed by RNA polymerase II into primary miRNAs (pri-miRNAs), which are then processed by Drosha complex into precursor miRNAs (pre-miRNAs), which are then exported into cytoplasm by the exportin 5 complex. Pre-miRNAs are then processed by the Dicer complex into mature miRNAs, which are further sorted into exosomes through four potential pathways namely: **a** nSMase2- dependent pathway; **b** 3′ end miRNA sequence-dependent pathway; **c** Sumoylated hnRNPs- dependent pathway; **d** miRISC related pathway. Exosomal miRNAs perform a functional role after being transported into recipient cells

According to current research, there are four possible routes for miRNAs to be sorted into exosomes, although their underlying mechanisms are yet unknown. The first sorting mechanism is the neural sphingomyelinase 2 (nSMase2)-dependent pathway. nSMase-2 molecule was the first to be correlated to miRNA sorting to exosomes. The proportion of exosomal miRNAs elevated when nSMase2 was overexpressed, whereas the proportion of exosomal miRNAs decreased when nSMase2 was inhibited [26]. The second sorting mechanism is the 3^ˈ^ end of the dependent pathway. The miRNA sequence of 3^ˈ^ end may carry a crucial sorting signal that aided in its inclusion into exosomes [27]. The sumoylated heterogeneous nuclear ribonucleoprotein (hnRNP)-dependent pathway is the third sorting mechanism. The three hnRNP family proteins are hnRNPA2B1, hnRNPA1, and hnRNPC among which hnRNPA2B1 could identify the GGAG pattern in the 3′ part of miRNA sequences and induce specific miRNAs to be packed into exosomes [28]. miRNA-induced silencing complex (miRISC) is the last sorting pathway. MVBs were discovered to co-localize with miRISC’s major components [29]. Thus, miRNAs have unique sequences that may direct their integration into exosomes. Figure 3 representing sorting mechanism of miRNAs into exosomes.

### Exosomal miRNAs in lung cancer

Lung cancer is one of the most common cancers in the world, as well as the leading cause of cancer-related death. Non-small cell lung cancer (NSCLC) is the main histological subtype of lung cancer, representing > 80% of lung cancer cases and includes adenocarcinoma, squamous-cell carcinoma, and large-cell carcinoma. Small cell lung cancer, represents the remaining 20% of lung cancer occurrences [30, 31]. Despite advancements in lung cancer treatment, patients’ prognosis is still poor. Therefore, early diagnosis of lung cancer before clinical manifestation may be an efficient method to decrease morbidity and mortality associated with the disease. Hence, exosomal miRNAs have been discovered to be novel biomarkers for lung cancer in several investigations.

#### Prognostic biomarker

Interestingly, evaluation of 103 NSCLC patients and 60 healthy controls revealed that serum exosomal miR-378 was highly overexpressed in NSCLC patients, and that this overexpression was correlated to positive lymph node metastasis and TNM advanced stage [32]. Overall, serum exosomal miR-378 holds great potential as a non-invasive prognostic biomarker for NSCLC screening and management [32]. Luo et al in 2021 explained the role of serum exosomal miR-382 in 126 NSCLC and 60 healthy patients. The exosomal miR-382 expression was evaluated using quantitative real time-polymerase chain reaction (RT-PCR) in all the individuals. NSCLC patients with lower serum exosomal miR-382 had a shorter overall survival (OS) rate. Thus, exosomal miR-382 appears to be a reliable prognostic biomarker for assessing NSCLC progression [33]. Kim et al 2021, investigated the significance of exosomal miR-1260b from NSCLC in tumor progression. Exosomal miR-1260b expression in plasma was higher in 48 NSCLC patients than in 48 healthy controls. Higher levels of exosomal miR-1260b were related to improved disease, metastasis, and poor survival. Therefore, exosomal miR-1260b may be a lung cancer prognostic marker [34]. Janpipatkul et al (2021), demonstrated ten exosomal miRNAs were markedly dysregulated in osimertinib-resistant NSCLC patients. Upregulation of all ten potential exosomal miRNAs was associated with a reduced risk of treatment failure and considerably better survival. Among them, four exosomal miRNAs (miR-323-3p, miR-1468-3p, miR-5189-5p and miR-6513-5p) were effectively increased and indicated the promising as a prognostic biomarker for accurately distinguishing osimertinib-resistant from osimertinib-sensitive NSCLC patients [35]. Further investigations are required on molecular mechanisms which would improve the treatment plans in lung cancer.

#### Diagnostic biomarker

Interestingly, Wu et al in 2020 assessed eight serum miRNAs and their correlating serum exosomal miRNAs (miR-21-5p, miR-141-3p, miR-126-3p, miR-146a-5p, miR-222-3p, miR-223-3p, miR-155-5p, and miR-486-5p) by using qRT-PCR in 48 healthy controls, 32 patients with lung benign lesion and 48 patients with early NSCLC at stages I/II. Their analysis suggested that serum exosomal miRNAs, rather than serum miRNAs, may be preferred biomarkers for patients with NSCLC in the early stages, and that a combination of serum miRNAs and serum exosomal miRNAs can help to enhance early NSCLC diagnosis [36]. Recently, Li et al and coworkers evaluated the levels of exosome-derived miR-3913-5p after the onset of resistance to osimertinib, an EGFR-targeted tyrosine kinase inhibitor. It was found that platelet count, tumor marker carcinoembryonic antigen, tumor, node and metastasis (TNM) stage and distant metastasis was associated to exosomal miR-3913-3P. Thus, exosomal miR-3913-5p in the peripheral blood of NSCLC patients could be utilized as diagnostic biomarkers for osimertinib resistance [37]. Altogether, the picture that emerges is that exosomal miRNAs are a promising class of biomarkers for NSCLC diagnosis though more research is needed for implantation in clinical practice.

#### Diagnostic and prognostic biomarkers

In 2020 Zhang et al screened for 22 exosomal miRNAs in which two exosomal miRNAs namely miR-125b-5p and miR-5684 were considerably down-regulated in 330 NSCLC patients compared to 312 healthy donors, indicating that (early) NSCLC has a high diagnostic efficiency. In addition, exosomal miR-125b-5p was correlated to metastasis, chemotherapeutic impact, and survival. Thus, the levels of exosomal miR-125b-5p and miR-5684 are considerably reduced in NSCLC patients, and they represent potential diagnostic and prognostic markers for the disease [38]. Tang et al (2020), demonstrated that when contrasted to 231 healthy controls, exosomal miR-620 levels were considerably lower in 235 NSCLC patients. Furthermore, downregulated exosomal miR-620 was related to chemotherapeutic action. These data points that serum exosomal miR-620 as a promising noninvasive prognostic and diagnostic biomarker in NSCLC patients [39]. Huang et al in 2020 investigated the levels of serum exosomal miR-1246 in 105 NSCLC patients. Importantly, serum exosomal miR-1246 was effective in discriminating NSCLC patients from healthy controls and patients with non-malignant respiratory illnesses. Furthermore, the amount of serum exosomal miR-1246 was substantially linked to TNM stage. Finally, serum exosomal miR-1246 has the potential to be a helpful diagnostic and prognostic marker for NSCLC [40]. In addition, Zheng et al 2021, reported six plasma exosomal miRNAs (miR-21, miR-214, miR-1246, miR-210, miR-96 and let-7 g) to differentiate radioresistant NSCLC patients from radiosensitive NSCLC patients, as well as to assess NSCLC prognosis. Among these candidates, plasma exosomal miR-96 and miR-1246, were demonstrated to be independent NSCLC diagnostic biomarkers in 52 patients with NSCLC compared to 45 healthy volunteers. More notably, miR-96 was found to be a promising diagnostic and prognostic biomarker for radioresistant NSCLC [41]. Further validation of above said identified exosomal miRNAs in lung cancer are warranted for accurate diagnosis and prognosis.

#### Therapeutic aspect

Mao et al (2021), identified the levels of exosomal miR-375-3p and was found to be substantially greater in 126 SCLC patients and also significantly connected with patients’ TNM stage. Hence, exosomal miR-375-3p has a great deal of promise as a new biomarker for monitoring metastasis and directing clinical treatment for SCLC patients [42]. Liu and co-workers investigated the exosomal miR-433 in the advancement of NSCLC and its mode of action. Exosomal miR-433 reduced tumor growth by interrupting the cell cycle and inducing apoptosis and T-cell infiltration in the tumor microenvironment of both in vivo and in vitro condition. Furthermore, miR-433 also decreased cisplatin resistance by controlling DNA damage. The existing findings could be used to develop a predictive prognostic biomarker and therapeutic target for NSCLC patients [43]. Exosomal miRNAs’ participation in many aspects of lung cancer will be revealed in the near future, and those prospective discoveries will surely improve our understanding of exosomal miRNAs in lung cancer and bring new insights in future diagnosis, prognosis, and therapy of lung cancer. Table 1 representing the exosomal miRNAs in biological fluids as prognostic and diagnostic biomarkers and therapeutic targets in lung cancer.

**Table 1:** Representing the exosomal miRNAs in biological fluids as prognostic and diagnostic biomarkers and therapeutic targets in lung cancer

Exosomal miRNAs	Sample Type	Role	Mechanism of action	References
miR-378	Serum	Prognostic	Screening and management of NSCLC	Zhang et al. (2020) [32]
miR-382	Serum	Prognostic	Improved the accuracy of carcinoembryonic antigen (CEA)	Luo et al. (2021) [33]
miR-1260b	Plasma	Prognostic	Promotes angiogenesis in HUVECs and metastasis of NSCLC	Kim et al. (2021) [34]
miR-323-3p, miR-1468-3p, miR-5189-5p and miR-6513-5p	Plasma	Prognostic	Distinguishing osimertinib-resistant from osimertinib-sensitive NSCLC patients	Janpipatkul et al. (2021) [35]
miR-486-5p and miR-146a-5p	Serum	Diagnostic	Improvement of early diagnosis for NSCLC.	Wu et al. (2020) [36]
miR-184, miR-3913-5p	Blood	Diagnostic	Indicate osimertinib resistance.	Li et al. (2021) [37]
miR-125b-5p and miR-5684	Serum	Diagnostic and Prognostic	Associated with metastasis, chemotherapeutic effect and survival	Zhang et al. (2020) [38]
miR-620	Serum	Diagnostic and Prognostic	Chemotherapeutic effect	Tang et al. (2020) [39]
miR-1246	Serum	Diagnostic and Prognostic	Associated with lymph node metastasis and TNM stage	Huang et al. (2020) [40]
miR-96	Plasma	Diagnostic and Prognostic	Radioresistant in NSCLC	Zheng et al. (2021) [41]
miR-375-3p	Blood	Therapeutic target	monitoring metastasis and guiding clinical therapeutics of SCLC patients	Mao et al. (2021) [42]
miR-433	Plasma	Therapeutic target	inhibits tumorigenesis through incremental infiltration of CD4 and CD8 cells	Liu et al. (2021) [43]

### Exosomal miRNAs breast cancer

Breast cancer is the most prevalent malignant tumor in women, and it poses a serious threat to women’s physical and mental health around the globe. Breast carcinoma is a heterogeneous molecular and morphological disease with three phenotypic grades and more than four distinct molecular subtypes at the gene expression level [44]. Breast cancer accounts for around a quarter of all cancer cases in women [45].

#### Prognostic biomarker

Li et al (2020), found that in patients with breast cancer, serum exosomal miR-148a levels are considerably lower. Serum exosomal miR-148a down-regulation was correlated to a poor clinical outcome in breast cancer patients. Hence, exosomal miR-148a in the serum might supply as an important biomarker as prognostic treatment of breast cancer [46]. In 2021, Xun and colleagues suggested that exosomes were used to transfer miR-138-5p from breast cancer cells to tumour-associated macrophages, which suppressed the expression of KDM6B, a protein coding gene. Thus, exosomes were involved in the transfer of miR-138-5p between cancer cells and macrophages, suggesting that circulating exosomal miR-138-5p could be used to establish a breast cancer prognostic indicator [47]. Wang and colleagues (2021) revealed that in breast cancer, plasma exosomal miR-363-5p acts as a tumour suppressor and also used as for non-invasive lymph node staging and prognosis prediction [48].

#### Diagnostic biomarker

In 2020, Wang et al reported that combination of the standard tumour marker carcinoma antigen 15–3 (CA15–3) and serum miR-1910-3p in exosomes was an efficient diagnostic marker that enhanced the reliability of breast cancer diagnosis. miR-1910-3p stimulated the NF-kB and wnt/−catenin signalling pathways, promoting breast cancer growth, by downregulating myotubularin-related protein 3. Thus, exosomal miR-1910-3p in serum could be a new molecular diagnostic for detecting breast cancer [49]. In addition, Lv et al (2020), suggested that exosomal miR-17-5p could discriminate between healthy and the breast cancer patients. Exosomal miR-17-5p outperformed traditional serum biomarkers CA125, and CA153 in terms of sensitivity and specificity and can be served as diagnostic biomarker [50]. Beyond serum, four exosomal urinary miRNAs (miR-423, miR-424, let7-i and miR-660) were found to be highly specific in distinguishing breast cancer patients from healthy individuals, with 100% selectivity and 98.6% sensitivity, by multilateral statistical analysis and can be used as diagnostic biomarker [45]. Liu et al (2021) discovered the plasma exosomal miR-21-5p was considerably up-regulated in breast cancer patients, which has diagnostic value and the specificity and sensitivity were 93.3 and 86.7% respectively. As a result, exosomal miR-21-5p in plasma could be employed as a biomarker in breast cancer diagnosis [51]. Li and co-workers (2021) demonstrated that high levels of miR-3662, miR-146a, and miR-1290 expression in the serum exosomes of breast cancer patients were correlated to lymph node metastases and breast cancer stage. Thus, exosomal miR-3662, miR-146a, and miR-1290 can be used as a promising diagnostic biomarker to clinically monitor breast cancer patients [52].

#### Therapeutic aspect

Interestingly, Ham et al in 2020 explained that exosomal miR-567 regulated autophagy and hence played a significant role in overcoming trastuzumab resistance, suggesting that exosomal miR-567 could be a promising therapeutic target and prognostic biomarker for breast cancer patients [53]. Jiang et al 2020 findings showed that tumor exosome-derived miR-9 and miR-181a stimulated the JAK/STAT signalling pathway by targeting SOCS3 and PIAS3 respectively, and thus promoted the expansion of early stage myeloid-derived suppressor cells (eMDSCs), indicating that exosomal-derived miR-9 and miR-181a could be used as a therapeutic target in the treatment of high IL-6 breast cancer [54]. Interestingly, in 2021 Shen et al reported that cancer-derived exosomal miR-7641 was contributed to understand the molecular pathways underlying cell–cell communication during breast tumour progression and metastasis, paving the way for better breast cancer diagnostic and therapeutic options [55]. The discovery of other exosomal miRNAs as appropriate biomarkers will provide unique insights and a more dynamic overview of the progression and therapeutic responses in breast cancer. Table 2 representing the exosomal miRNAs in biological fluids as prognostic and diagnostic biomarkers and therapeutic targets in breast cancer.

**Table 2:** Representing the exosomal miRNAs in biological fluids as prognostic and diagnostic biomarkers and therapeutic targets in breast cancer

Exosomal miRNA	Sample Type	Role	Mechanism of action	Reference
miR-148a	Serum	Prognosis	Tumor-node-metastasis (TNM) stage, differentiation, and lymph node metastasis	Li et al. (2020) [46]
miR-138-5p	Serum	Prognosis	modulates polarization of tumor-associated macrophages	Xun et al. (2021) [47]
miR-363-5p	Plasma	Prognostic	inhibits lymph node metastasis by downregulating PDGFB	Wang X et al. (2021) [48]
miR-1910-3p	Serum	Diagnostic	promotes proliferation, metastasis, and autophagy of breast cancer cells	Wang B et al. (2020) [49]
miR-17-5p	Serum	Diagnostic	Predicted target genes	Lv et al. (2020)
miR-423, miR-424, let7-i and miR-660	Urine	Diagnostic	Tumor suppressor effect	Hirschfeld et al. (2020) [45]
miR-21-5p	Plasma	Diagnostic	Tumor suppressor effect	Liu et al. (2021) [51]
miR-146a, miR-1290 and miR-3662	Serum	Diagnostic	monitor patient condition in the course of surgery and chemotherapy.	Li et al. (2021) [52]
miR-567	Tissue and serum	Therapeutic target	Reversing trastuzumab resistance via regulating autophagy	Han et al. (2020) [53]
miR-9 and miR-181a	Blood	Therapeutic target	promoted the expansion of eMDSCs	Jiang et al. (2020) [54]
miR-7641	Plasma	Therapeutic target	promotes breast cancer progression and metastasis	Shen et al. (2021) [55]

### Exosomal miRNAs in prostate cancer

Prostate cancer is the most frequent cancer in males, and it is also the second highest cause of death in men after lung cancer [3]. Prostate specific antigen (PSA), a protein released primarily by prostate cells, has been utilised as a blood-based biomarker for prostate cancer for decades. PSA is a useful tool; however, it lacks specificity and is thus not regarded as an ideal biomarker [56]. As a result, novel and specific prostate cancer markers are desperately needed.

#### Prognostic biomarker

Guo et al validated a cohort of 108 treatment-naive prostate cancer and 42 castration resistance prostate cancer (CRPC) patients and found differential expression of six plasma exosomal miRNAs (miR-423-3p, miR-320d, miR-99a-5p, miR-320b, miR-150-5p and miR-320a). In which exosomal miR-423-3p was specifically linked with CRPC. Therefore, exosomal miR-423-3p might act as prognostic biomarker for early identification and prediction of castration resistance [57]. A screen of 21 urinary exosomal miRNAs to differentiate between the biochemical recurrence (BCR) patients and non-BCR patients was performed recently be Kim and colleagues. BCR is explained as increasing serum PSA levels following radical prostatectomy. Three miRNAs (miR-532-5p, miR-26a-5p and miR-99b-3p) were increased in exosomes from BCR patients in a validation study. Finally, it was concluded that urinary exosomal miR-532-5p would be used as a prognostic biomarker to predict BCR [58]. One recent study also identified 14 exosomal miRNAs as candidate for specific non-invasive biomarkers in metastatic prostate cancer (mPCa) in plasma. Five miRNAs were validated in mPCa exosomes, confirming that miR-205-5p, miR-183-5p, miR-425-5p, miR-148a-3p, and miR-125b-5p were differentially expressed. Out these, exosomal miR-425-5p showed a potential value as prognostic biomarker for mPCa [59]. Shin et al (2021) investigated exosomal miRNA expression in urine samples of 149 prostate cancer and identified five exosomal miRNAs related with metastasis: miR-16-5p, miR-451a, miR-142-3p, miR-21-5p and miR-636. These findings suggested that urinary exosomal miRNAs could be used as non-invasive markers to predict metastasis and prognosis in prostate cancer patients [60]. Altogether, these studies suggest that exosomal miRNAs in urine samples might be a non-invasive marker for prognosis in the patients with prostate cancer.

#### Diagnostic biomarker

Exosomal miR-141-5p levels in prostate cancer patients’ plasma were higher than in healthy controls, but miR-125a-5p levels were significantly lower in patients with prostate cancer than in healthy controls. Therefore, the findings imply that high miR-141-3p expression and low miR-125a-5p expression in plasma exosomes from prostate cancer patients could be helpful diagnostic biomarkers of certain tumour characteristics linked with prostate cancer [61]. A recent paper by Li et al reported that urinary exosomal miR-375 expression was significantly downregulated in prostate cancer patients compared to healthy individuals, whereas urinary exosomal miR-486-5p, miR-451a and miR-486-3p expression levels were up-regulated. Urine exosomal miR-375 was effective in distinguishing between localised and metastatic prostate cancer. Thus, the urinary exosomal miRNAs have potential as non-invasive biomarkers for diagnosing and predicting prostate cancer progression [62].

#### Therapeutic aspect

Interestingly, Che et al in 2019 investigated that exosomal-miR-143 was able to decrease the levels of proliferating cell nuclear antigen (PCNA), matrix metalloproteinase (MMP)-2 and MMP-9 expression as well as PC3 cell proliferation, migration, invasion and tumor formation, whereas enhanced apoptosis. Finally, exosomal miR-143 suppressed prostate cancer by directly and adversely targeting TFF3. Hence, it was suggested the exosomal-miR-143 can be used as a therapeutic target in treating prostate cancer [63]. Yu et al in 2021 explained that exosomal miR-92a-1-5p was able to suppress type I collagen production by directly targeting COL1A1, thus promoting osteoclast development and preventing osteoblastogenesis. Thus, suggesting that exosomal miR-92a-1-5p could be a potential therapeutic target for prostate cancer bone metastasis [64]. Zhou et al (2020) studies on in vitro and in vivo conditions explained that exosomal miR-217 levels boosted cell proliferation and invasion, whereas exosomal miR-23b-3p levels inhibited cell proliferation and invasion. Thus, the epithelial-mesenchymal transition process may have influenced this regulation, suggesting that exosomal miR-217 and miR-23b-3p could be exploited as potential targets in the diagnosis and therapy of prostate cancer [65]. In addition, Shan G et al. (2020) findings showed that cancer-associated fibroblasts secreted exosomal miR-423-5p increased chemotherapy resistance by targeting GREM2 via the TGF-β pathway. This research could lead to the development of new therapeutic approach in prostate cancer [66]. Exosome-specific investigations on developing prostate cancer diagnostics and novel treatment targets are very promising and further research is required to prove in clinical use. Table 3 representing the exosomal miRNAs in biological fluids as prognostic and diagnostic biomarkers and therapeutic targets in prostate cancer.

**Table 3:** Representing the exosomal miRNAs in biological fluids as prognostic and diagnostic biomarkers and therapeutic targets in prostate cancer

Exosomal miRNA	Sample Type	Role	Mechanism of action	Reference
miR-423-3p	Plasma	Prognostic	Early detection/prediction of castration-resistance.	Guo et al. (2021) [57]
miR-532-5p	Urine	Prognostic	important predictive factor for BCR of intermediate-risk of prostate cancer	Kim et al. (2021) [58]
miR-425-5p	plasma	Prognostic	enrichment of genes related to bone development	Rode et al. (2021) [59]
miR-16-5p, miR-451a, miR-142-3p, miR-21-5p and miR-636	Urine	Prognostic	for predicting metastasis	Shin et al. (2021) [60]
miR-125a-5p and miR-141-5p	Plasma	Diagnostic	Tumor suppressor	Li et al. (2020) [61]
miR-375 and miR-451a	Urine	Diagnostic	predicting the progression of PCa.	Li et al. (2021) [62]
miR-143	Prostate cancerous tissue	Therapeutic target	inhibited the expression ofPC3 cell proliferation, migration, invasion, and tumor growth	Che et al. (2019) [63]
miRNA-92a-1-5p	Serum	Therapeutic target	PCa bone metastasis.	Yu et al. (2021) [64]
miR-423-5p	Cancerous cells	Therapeutic target	promotes chemotherapy resistance in prostate cancer by targeting GREM2 through the TGF-β signaling pathway	Shan et al. (2020) [66]

### Exosomal miRNAs in Oral squamous cell carcinoma (OSCC)

OSCC is the world’s sixth most common cancer, and it affects the tongue, lip, buccal mucosa, mouth floor, hard palate, retromolar trigone and gingiva [67]. Despite tremendous advancements in therapy strategies over the past decades, the prognosis for OSCC has not improved significantly. The biggest problems with OSCC are tumor development and recurrence [68], thus warranting the identification of novel biomarkers for tumor progression.

#### Diagnostic and prognostic biomarker

Interestingly, as an alternative to blood or urine, salivary exosomal miR-24-3p has shown potential for OSCC diagnosis. Exosomal miR-24-3p promoted the growth of malignant cells and also overexpression of exosomal miR-24a-3p aided OSCC cell proliferation through regulating the expression of cell cycle-related genes. Moreover, exosomal miR-24a-3p can inhibit OSCC cells from proliferating by targeting PER1. Thus, exosomal miR-24a-3p could be a new OSCC diagnostic biomarker as well as a therapeutic target [69]. He et al (2021) found that the plasma exosomal miR-130a were substantially elevated in 184 OSCC patients than in 196 healthy controls. Exosomal miR-130a was also found to be an independent predictive factor for overall survival and recurrence-free survival. Thus, exosomal miR-130a could be a viable diagnostic and prognostic biomarker in treating OSCC [70]. Chen et al (2021) demonstrated that in primary OSCC cells and OSCC patients’ serum, exosomal miR-155 and miR-21 were considerably increased, while exosomal miR-126 was dramatically downregulated. Moreover, exosomal miR-155 and miR-21 are oncogenic miRNAs that decrease the expression of PTEN and Bcl-6. Exosomal miRNAs (miR-155 and miR-21) could thus be used as biomarkers for OSCC diagnosis and prognosis [71]. Noteworthy, that there are only few studies have been performed to prove that exosomal miRNAs as a diagnostic and prognostic biomarker in OSCC. So, more validated research is required to prove the state of the disease using exosomal miRNAs.

#### Therapeutic aspect

Fascinatingly, The AKT/GSK-3/β-catenin/Snail signaling cascade confers aggressiveness in oral cancer cells through the exosomal miR-34a-5p/AXL axis, which could be a therapeutic target for OSCC [72]. Sun et al (2019) revealed that exosomal miR-382-5p overexpression was found in cancer associated fibroblasts (CAFs) as compared to fibroblasts from normal tissue, thus responsible for OSCC cell migration and invasion. This work established a new method of CAF-assisted OSCC advancement, which could aid in the discovery of new cancer treatment targets [73]. Kulkarni et al (2020) findings demonstrated the importance of exosomal-mediated miR-30a transfer in restoring cisplatin-resistant OSCC cells via Beclin 1 and Bcl2 regulation, implying a possible therapeutic role [74]. Kirave et al (2020) findings emphasized the importance of exosomal-mediated miR-155 shuttling in the cisplatin-chemoresistance shown in OSCC cells, indicating that miR-155 signalling could be targeted for oral cancer therapy [75]. Exosomes delivered miR-130b-3p to human umbilical vein endothelial cells (HUVECs), which increased angiogenesis and inhibited PTEN expression. Exosome-mediated miR-130b-3p has been shown to enhanced progression and tubular development in OSCC. These findings suggest that it could be used as prognostic biomarker or a therapeutic target for OSCC treatment [76]. Yuan et al (2021) results indicated that the tumor suppressor LATS2 gene is suppressed by M2 macrophage-derived exosomal miR-31-5p, which may provide new targets for OSCC molecular therapy by blocking the Hippo signalling pathway and facilitating the growth of OSCC [77]. Table 4 representing the exosomal miRNAs in biological fluids as prognostic and diagnostic biomarkers and therapeutic targets in oral squamous cell carcinoma.

**Table 4:** Representing the exosomal miRNAs in biological fluids as prognostic and diagnostic biomarkers and therapeutic targets in oral squamous cell carcinoma

Exosomal miRNA	Sample Type	Role	Mechanism of action	Reference
miR-24-3p	Saliva	Diagnostic	inhibit OSCC cells from proliferating by targeting PER1	He et al. (2020) [69]
miR-130a	Plasma	Diagnostic and prognostic	predictive factor for overall survival and recurrence-free survival	He et al. (2021) [70]
miR-155 and miR-21; miR-126	Blood	Diagnostic and prognostic	suppress PTEN and Bcl-6 expression; tumor suppressor	Chen et al. (2021) [71]
miR-34a-5p	Primary fibroblasts	Therapeutic target	enhanced nuclear translocation of β-catenin	Li et al. (2018) [72]
miR-382-5p	Tissue and fibroblast cell	Therapeutic target	promotes the migration and invasion	Sun et al. (2019) [73]
miR-30a	Serum	Therapeutic target	regaining sensitivity of the cisplatin-resistant	Kulkarni et al. (2020) [74]
miR-130b-3p	Serum	Therapeutic target	Promotes Progression and Tubular Formation	Yan et al. (2021) [76]

### Exosomal miRNAs in colorectal cancer

Exosomal miRNAs have also garnered attention in recent years for detection, prognosis and treatment of colorectal cancer (CRC), one of the most frequent malignancies in the globe, with a significant mortality rate among those discovered late in the disease’s progression [78].

#### Diagnostic biomarker

Li et al 2020 found reduced expression level of exosomal miR-139-3p in CRC patients’ plasma may serve as a potential biomarker for early diagnosis and monitoring of metastasis [79]. Notably, (miR-126, miR-1290, miR-23a, and miR-940) four exosomal miRNAs in CRC patient serum that could distinguish CRC at TNM stage I from healthy controls. Thus, these exosomal miRNAs proved to be a promising diagnostic biomarker in CRC [80]. Handa et al (2021) found seven exosomal miRNAs (miR-21-5p, miR-1246, miR-1268a, miR-1290, miR-4284, miR-4323 and miR-6766-3p) were overexpressed in colorectal adenoma (CRA). However, in post-treatment sera, four of these exosomal miRNAs (miR-1246, miR-1290, miR-4323, and miR-4284) were considerably lower in CRA organoid culture. Thus, the expression of these four exosomal were discovered to represent promising CRA diagnostic biomarkers [81].

#### Diagnostic and prognostic biomarker

Liu et al (2020) demonstrated that CRC patients with metastasis had elevated serum exosomal miR-106b-3p expression compared to those without metastasis, and might therefore be a promising prognostic biomarker and therapeutic target [82]. Analysis of 125 colorectal cancer patients, 70 healthy controls and 45 benign adenomas found serum exosomal miR-874 was considerably downregulated in the CRC patients. The expression of serum exosomal miR-874 was discovered to be a statistically significant independent predictive factor for CRC patients’ overall survival. Thus, exosomal miR-874 expression in serum could be a useful biomarker for CRC diagnosis and prognosis [83]. The expression of exosomal miR-122 in serum was considerably elevated in CRC patients, particularly those with liver metastasis. The serum exosomal miR-122 was presented to be an independent predictive biomarker of CRC patients using univariate and multivariate logistic regression [84]. Exosomal let-7 g and miR-193a isolated from patient plasma were also identified as cancer progression markers. Moreover, low miR-193a and high let-7 g expression had a lower survival rate and might be used as a marker for CRC diagnosis and prognosis purpose [85].

#### Therapeutic aspect

Liu et al (2020) demonstrated that CRC patients with metastasis had elevated serum exosomal miR-106b-3p expression compared to those without metastasis, and might therefore be a promising prognostic biomarker and therapeutic target [82]. Tian et al (2021) showed that CRC, exosomes containing miR-221/222 activate hepatic hepatocyte growth factor (HGF) through reducing SPINT1 expression in an in vitro cell co-culture model. Exosomal miR-221/222 may increase CRC progression and suggesting that it might be used as a new prognostic marker and therapeutic target [86]. All these studies provide intriguing evidence for exosomal miRNAs and their potential significance as CRC biomarkers. But however, there are inadequate studies to prove exosomal miRNAs as biomarkers for CRC prognosis and diagnosis and therapeutic aspect to explore in clinical studies. Table 5 representing the exosomal miRNAs in biological fluids as prognostic and diagnostic biomarkers and therapeutic targets in colorectal cancer.

**Table 5:** Representing the exosomal miRNAs in biological fluids as prognostic and diagnostic biomarkers and therapeutic targets in colorectal cancer

Exosomal miRNA	Sample Type	Role	Mechanism of action	Reference
miR-139-3p	plasma	Diagnosis	monitoring of metastasis	Li et al. (2020) [46]
miR-126, miR-1290, miR-23a, and miR-940	Serum	Diagnostic	distinguish CRC at TNM stage I	Shi et al. (2021) [80]
miR-4323, miR-4284, miR-1290, and miR-1246	Serum	Diagnostic	CRA organoid culture	Handa et al. (2021) [81]
miR-106b-3p	Serum	Diagnostic and prognostic	promotes metastasis by down-regulating DLC-1 expression.	Liu et al. (2020) [82]
miR-874	Serum	Diagnostic and prognostic	positive lymph node metastasis, poor differentiation, and advanced TNM stage.	Zhang et al. (2020) [83]
let-7 g and miR-193a	Plasma	Diagnostic and prognostic	Accelerated cancer progression	Cho et al. (2021) [85]
miR-106b-3p	Serum	Therapeutic target	promotes metastasis by down-regulating DLC-1 expression	Liu et al. (2020) [82]
miR-221/222	Serum	Therapeutic target	exacerbates tumor liver metastasis by targeting SPINT1	Tian et al. (2021) [86]

### Other exosomal non-coding RNAs in various cancers

Non-coding RNAs (ncRNAs) interact with other nucleic acids and proteins in intricate networks that have far-reaching consequences for cell biology [87]. ncRNAs are distributed into various types namely long non-coding RNAs (lncRNAs), miRNAs, short non-coding RNAs (sncRNAs), small nuclear RNAs (snRNAs) and small nucleolar RNAs (snoRNAs) etc. [88]. Interestingly, exosomes can also include lncRNAs, which operate as messengers in cell-to-cell interactions. Importantly, some lncRNAs are abundant in exosomes while others are limited, implying that some lncRNAs are sorted into exosomes selectively. lncRNAs originating from exosomes have recently been found to influence tumour apoptosis, proliferation and migration, as well as promote angiogenesis [89]. These lncRNAs produced from exosomes have the potential to be used as diagnostic and prognostic biomarkers for various cancers.

Importantly, Rao et al, (2019) discovered a link between HAGLR downregulation and a higher circulating tumor cells (CTCs) detection rate in NSCLC patients with a poor prognosis. In this study, plasma exosomes from 40 NSCLC patients were extracted, and HAGLR expression levels were compared by qRT-PCR to that of eight healthy controls. HAGLR levels were shown to be lower in patients who were in later stages of cancer and had shown poor prognosis [90]. Lu et al., (2021) stated that in vitro, exosomal LINC00662 aided the progression of NSCLC via the miR-320d/E2F1 axis and in vivo, exosomal LINC00662 increased tumor growth in NSCLC. Thus, this finding gave new light on how exosomal lncRNA LINC00662 plays a role in the progression of NSCLC and may serve as a potential target [91].

Interestingly, Zhong et al, (2020) study stated that exosomal H19 expression was elevated in breast cancer patients compared to benign breast disease patients and the healthy controls. Furthermore, levels of exosomal H19 expression were related to lymph node metastasis, TNM stages and distant metastasis. Thus, the overall results indicated that serum exosomal H19 could be a novel biomarker for breast cancer diagnosis [92]. Wang et al, (2019) explained that exosomal circulatory HOTAIR was found in breast cancer patients and used to investigate the disease pathology. HOTAIR RNA in exosome was detected by using qRT-PCR. The state of the receptor tyrosine kinase (RTK) ErbB2 (commonly known as HER2/neu) in tumor tissues was positively linked with exosomal HOTAIR expression. This research provided a biological foundation for the development of novel liquid biopsy biomarkers and targeted therapeutics for malignant breast cancer with greater precision [93].

Furthermore, Jiang et al, (2021) reported that the exosomal lncRNA HOXD-AS1 was found to be elevated in exosomes produced from castration-resistant prostate cancer (CRPC) cell lines and serum exosomes from metastatic prostate cancer patients, which associated with tissue expression. Prostate cancer cells actively absorbed exosomal HOXD-AS1, which acted as competing endogenous RNA (ceRNA) to alter the miR-361-5p/FOXM1 axis, driving prostate cancer metastasis. This study provided novel insights into the regulation of prostate cancer distant metastasis by the exosomal HOXD-AS1 mediated miR-361-5p/FOXM1 axis, as well as a promising liquid biopsy biomarker for detecting and treating metastatic prostate cancer [94]. Zhou et al, (2021) investigations revealed that exosomal lnRNA ADAMTS9-AS2 inhibited OSCC cell proliferation, migration, and invasion in vitro condition. These results suggested the critical role of ADAMTS9-AS2 in the cell microenvironment during OSF carcinogenesis, and it is predicted to be used as a marker for OSCC early detection [95]. Hu et al in 2018 stated that exosomes were separated from the plasma of CRC patients (*n* = 50) and healthy people (n = 50), and total exosomal RNAs were extracted using the TRIzol reagent. The results indicated that six lncRNAs (LNCV6 116,109, LNCV6 98,390, LNCV6 38,772, LNCV 108266, LNCV6 84,003, and LNCV6 98,602) were shown to have considerably higher expression in CRC patients as compared to healthy people and may serve as potential non-invasive biomarkers for early diagnosis of CRC treatment [96]. In general, exosomal lncRNAs, circRNAs and piRNAs are involved in the tumor development. However, there are limited publications on the role of exosomal ncRNAs in OSCC function and their potential as biomarkers, both of which demand additional attention and investigation.

### Future perspective and clinical relevance

Early cancer detection is critical for extending life expectancy and reducing disease-related mortality. Exosomes have been a centre for investigation as disease biomarkers. The first global cancer diagnostic device based on exosomes was launched in the United States on January 21, 2016. Exosome Diagnostics’ liquid biopsy products were a pivotal event in exosomal biology [97]. Exosomal miRNAs can give stable, sensitive, and precise biological information as a biomarker. However, whether exosomal and free miRNAs have different diagnostic and prognostic activities is less clear, nor is it known if exosomal and free miRNAs are controlled differently in response to specific stimuli.

The key benefits of liquid biopsy include high specificity, a non-invasive method, and the ability to repeat the procedure to allow real-time patient monitoring. Exosome-based research currently confronts a few challenges, including the multistep process necessary from serum collection to miRNA measurement. This is a limitation when compared to emerging cancer biomarker detection technologies, some of which involve the observation of miRNAs or other markers directly in whole blood or serum without the need for additional processing, and hence may be more easily integrated into the clinical context [98].

Despite many advantages of exosomal miRNAs in cancer diagnosis, there are still a few challenges that are to be resolved. To begin with, biological body fluids are a rich source of exosomes from several sources, which results in complicating the isolation of tumor cell-derived exosomes. Second, despite the fact that several isolation strategies have been offered, the purity and quality of exosomes varies depending on the techniques, and there is still no standard methodology for exosome separation and identification.

Future research must determine whether miRNA packaging into exosomes and exosomal absorption is a selective or stimulus-dependent mechanism. As a result, using exosomal miRNAs in therapeutic treatment is a difficult yet fascinating task that scientists and practitioners should investigate more. Despite these obstacles, it is critical that researchers continue to work on determining the clinical value of exosomal miRNAs as cancer biomarkers so that they might improve patient outcomes.

## Conclusion

The lack of appropriate and usable biomarkers for cancer diagnosis and prognosis adds to cancer patients’ dismal survival rates. Liquid biopsy is an innovative method in clinical oncology that has the ability to screen and diagnose patients with a variety of cancers. As a result, liquid biopsy may play a role in guiding clinicians in cancer treatment in the future. As liquid biopsy biomarkers, exosomal miRNAs have the potential to provide non-invasive insight to guide disease management. The effectiveness of liquid biopsies in the clinical setting will require more multi-centre, larger-scale, and longer-term investigations. Thus, exosomal miRNAs are intriguing liquid biopsy biomarker candidates that could improve the prognostic and diagnostic profiles of cancer and other diseases.

## Data Availability

Not applicable.

## Abbreviations

ALIX: ALG-2-interacting protein X
BCR: Biochemical recurrence
CA15–3: Carcinoma antigen 15–3
CRA: Colorectal adenoma
CRC: Colorectal cancer
CRPC: Castration resistance prostate cancer
ESCRT: Endosomal sorting complex required for transport
HGF: Hepatocyte growth factor
hnRNP: heterogeneous nuclear ribonucleoprotein
ILVs: Intraluminal vesicles
miRISC: miRNA-induced silencing complex
miRNAs: microRNAs
mPCa: metastatic prostate cancer
mRNAs: messenger RNAs
MVBs: Multivesicular bodies
ncRNAs: non-coding RNAs
NSCLC: Non-small cell lung cancer
nSMase2: neural sphingomyelinase 2
OS: Overall survival
OSCC: Oral squamous cell carcinoma
RT-PCR: Real time-polymerase chain reaction
TNM: Tumor, node and metastasis
TSG101: Tumour susceptibility gene 101
